# Identification of Potential Inhibitors of 3CL Protease of SARS-CoV-2 From ZINC Database by Molecular Docking-Based Virtual Screening

**DOI:** 10.3389/fmolb.2020.603037

**Published:** 2020-12-17

**Authors:** Ashraf Ahmed Ali Abdusalam, Vikneswaran Murugaiyah

**Affiliations:** ^1^Department of Pharmaceutical Sciences, Faculty of Health Sciences, Sirte University, Sirte, Libya; ^2^Discipline of Pharmacology, School of Pharmaceutical Sciences, Universiti Sains Malaysia, Penang, Malaysia

**Keywords:** virtual screening, docking, ZINC database, COVID-19, SARS-CoV-2, 3CL protease

## Abstract

The rapid outbreak of Coronavirus Disease 2019 (COVID-19) that was first identified in Wuhan, China is caused by a novel severe acute respiratory syndrome coronavirus 2 (SARS-CoV-2). The 3CL protease (3CLpro) is the main protease of the SARS-CoV-2, which is responsible for the viral replication and therefore considered as an attractive drug target since to date there is no specific and effective vaccine available against this virus. In this paper, we reported molecular docking-based virtual screening (VS) of 2000 compounds obtained from the ZINC database and 10 FDA-approved (antiviral and anti-malaria) on 3CLpro using AutoDock Vina to find potential inhibitors. The screening results showed that the top four compounds, namely ZINC32960814, ZINC12006217, ZINC03231196, and ZINC33173588 exhibited high affinity at the 3CLpro binding pocket. Their free energy of binding (FEB) were −12.3, −11.9, −11.7, and −11.2 kcal/mol while AutoDock Vina scores were −12.61, −12.32, −12.01, and -11.92 kcal/mol, respectively. These results were better than the co-crystallized ligand N3, whereby its FEB was −7.5 kcal/mol and FDA-approved drugs. Different but stable interactions were obtained between the four identified compounds with the catalytic dyad residues of the 3CLpro. In conclusion, novel 3CLpro inhibitors from the ZINC database were successfully identified using VS and molecular docking approach, fulfilling the Lipinski rule of five, and having low FEB and functional molecular interactions with the target protein. The findings suggests that the identified compounds may serve as potential leads that act as COVID-19 3CLpro inhibitors, worthy for further evaluation and development.

## Introduction

Coronavirus disease (COVID-19) began in the Hubei Province of China in late 2019 (World Health Organization [WHO], 2020a), and caused by a novel severe acute respiratory syndrome coronavirus 2 (SARS-CoV-2). Being highly infectious, this virus poses a grave threat to the global populations associated with a high rate of mortality (Granlinski and Menachery, 2020; Wu et al., 2020; Zhao et al., 2020). Symptoms linked with this disease include fever, myalgia, cough, dyspnea and fatigue (Huang et al., 2020 and Jin et al., 2020a). On 30 Jan 2020, the outbreak was declared by the World Health Organization (WHO) as a Public Health Emergency of International Concern, while on 11 March 2020, WHO has declared the COVID-19 outbreak a global pandemic (Rodríguez-Morales et al., 2020; World Health Organization [WHO], 2020b). Currently, there is still no treatment available for COVID-19 and investigations concerning the treatment of this infection is actively ongoing, especially vaccines (Li and De Clercq, 2020; Rodríguez-Morales et al., 2020). Nevertheless, treatments with well-known drugs such as chloroquine or investigational drug such as remdesivir are suggested for this disease (Colson et al., 2020; Touret and de Lamballerie, 2020; Wang et al., 2020). Cocktail of human immunodeficiency virus (HIV) drugs, lopinavir/ritonavir is also being investigated as a therapy for COVID-19 as they exhibited anti-coronavirus effect *in vitro* (Que et al., 2003; Chu et al., 2004; Chan et al., 2015; Li and De Clercq, 2020).

The SARS-CoV-2, belonging to beta-coronavirus that originated from bats has an envelope and sense single-stranded RNA (Perlman and Netland, 2009; Cui et al., 2019). The virus contains four non-structural proteins: papain-like (PLpro) and 3-chymotrypsin-like (3CLpro) proteases, RNA polymerase and helicase (Zumla et al., 2016). Both proteases (PLpro and 3CLpro) are involved with transcription and replication of the virus. Amongst the four types, the 3CLpro is considered to be mainly involved in the replication of the virus (de Wit et al., 2016). A study reported that the main protease 3CLpro of COVID-19 showed 96% sequence similarity with that of SARS-CoV (Xu et al., 2020).

The adoption of computational methods has been applied in the process of drug discovery, which helped to speed up discovery and design of new drug candidates at a lower cost (Zoete et al., 2009). Virtual screening-based drug discovery is recognized as one of the efficient strategies that may help in the field of invention and development of new drugs (Sliwoski et al., 2014). Virtual screening (VS) is a widely used computational approach that evaluates the potential drug candidates *in silico*. It is used to find different molecular scaffolds that act on a target protein of interest in the process of discovering chemical starting points as novel or potential leads for further optimization and development as alternatives to clinically available drugs. The method employs sequential filters, thus a large number of compounds could be screened to identify the potential lead-like hits for further biological evaluation on drug target *in vitro* and *in vivo* (Jacq et al., 2007; McInnes, 2007; Lavecchia and Di Giovanni, 2013). There are many free databases that offers selection of compounds for VS, one such database is the ZINC database that has 35 millions compounds. These compounds are also available for purchase. The database also provides information on the chemical and physical properties of the compounds such as molecular weight, log P, number of hydrogen-bond donor and acceptors, types of bonds and many others (Irwin et al., 2012).

The present study aimed to apply the VS approach to identify potential COVID-19 3CL protease inhibitors retrieved from the ZINC database and FDA-approved drugs, followed by molecular docking analysis to discover novel inhibitors that could be used as potential leads for treatment of coronavirus related infection.

## Materials and Methods

### Sequence Alignment

For determination of the conserved functional residues between the two proteins, 6LU7 has a resolution of 2.16 Å for COVID-19 (Jin et al., 2020b) and 2A5I has a resolution of 1.88 Å for SARS-CoV (Lee et al., 2005), a multiple sequence alignment analysis was performed, which can be used as potential targets for the discovery of drug hits. Both proteins were retrieved from the protein data bank (PDB) in three-dimensional structures and the sequence was generated using discovery studio software.

### Preparation of Protein for Docking

The crystal structure of the 3CL main protease in complex with a peptide-like inhibitor N3 was obtained from the Protein Data Bank (PDB ID: 6LU7) (Burley et al., 2017). The co-factor and water molecules were removed, and hydrogen was added using AutoDockTools (ADT).

### Screening of ZINC Database Ligand Molecules

The three-dimensional structures of 2000 ligand molecules used in this study were obtained from the download page of the ZINC database, by using the multiple options available the datasets were downloaded (Irwin and Shoichet, 2005) in mol2 format. The ligands were converted to PDBQT (Forli et al., 2016) for VS with AutoDock Vina. Molecular properties were derived from the ZINC website; the purpose was to assess the likelihood of the molecules to have drug-like properties.

### Virtual Screening and Molecular Docking

Virtual screening was performed using the AutoDock Vina (Trott and Olson, 2010). The files used include protein converted from pdb to pdbqt and Config.txt file created including all the information required for VS using ADT, other configurations were considered a default. AutoDock 4.2 (Morris et al., 2009) was used during the docking process; the center grid parameter was specified as 60-60-60 for *x*-, *y*- and *z*-axes, respectively, with a spacing of 0.375 Å and located at the center of the active site. One hundred independent runs were carried out for each docking experiment. The lowest energy of binding was selected for each conformation.

## Results and Discussion

### Analysis of Sequence Alignment Among Two Coronaviruses

**FIGURE 1:**
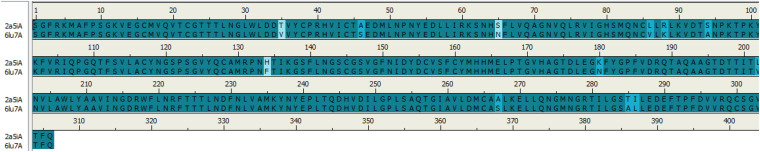
Sequence alignment of COVID-19 3CLprotease and SARS-CoV. The image was generated by Discovery studio.

Sequence alignments of SARS-CoV and COVID-19 3CL protease are displayed in Figure 1; the number of amino acids residues was identical beginning from Ser1 to Gln306. The sequence alignment of the two proteins COVID-19 (PDB:6LU7) and SARS-CoV (PDB:2A5I) were similar with 96%, and the differences were at twelve positions in the sequence alignment.

**FIGURE 2:**
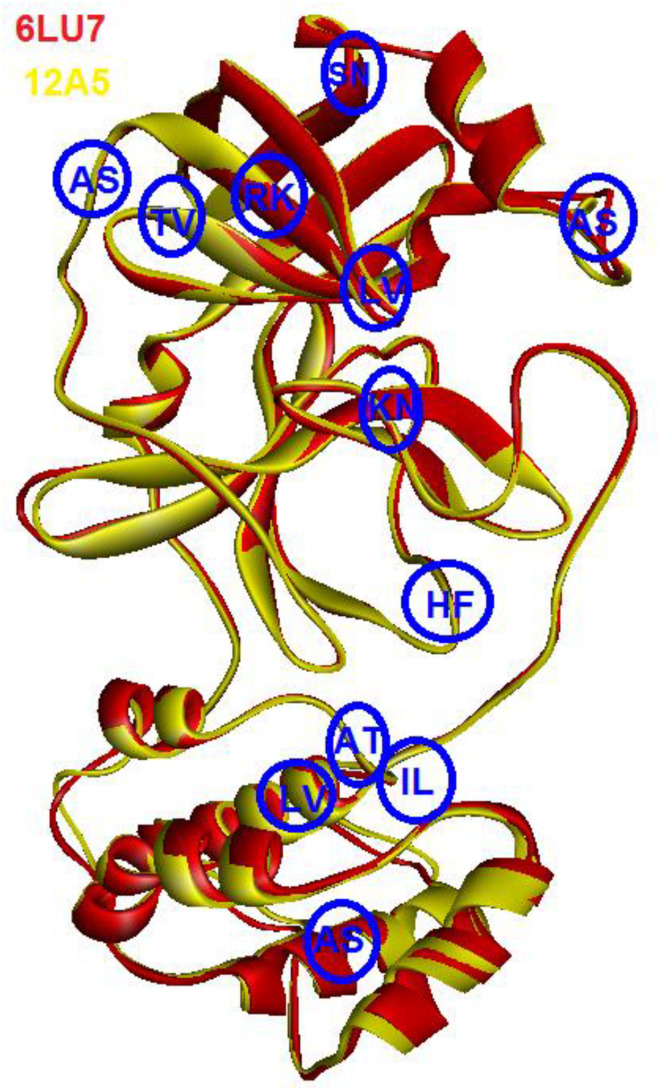
Superimposed of COVID-19 3CLprotease (red) to SARS-CoV (yellow). The blue circle showed the position of different amino acids between the two proteins. The letters indicate the code of the amino acid.

As can be seen in Figure 2, the 3D structures of superimposed SARS-CoV and COVID-19 3CL protease showed differences in twelve amino acids, whereby their α carbon atoms are of at least 1 nm away from the binding pocket. Additionally, the obtained results exhibited that COVID-19 3CL protease has a Cys-His catalytic dyad (Cys145 and His41) consistent with SARS CoV 3CLpro (Cys-145 and His-41) (Yang et al., 2003). Besides, the alignment and superimposing of the two coronaviruses explained that the conserved catalytic dyad residues Cys145 and His41 existing precisely at similar location in the binding pocket. The results of the analysis of the sequence and structural alignment proved that conserved functional residues exist within the binding pockets of amongst COVID-193CLprotease and SARS-CoV.

### Validation of the Virtual Screening Protocol

**FIGURE 3:**
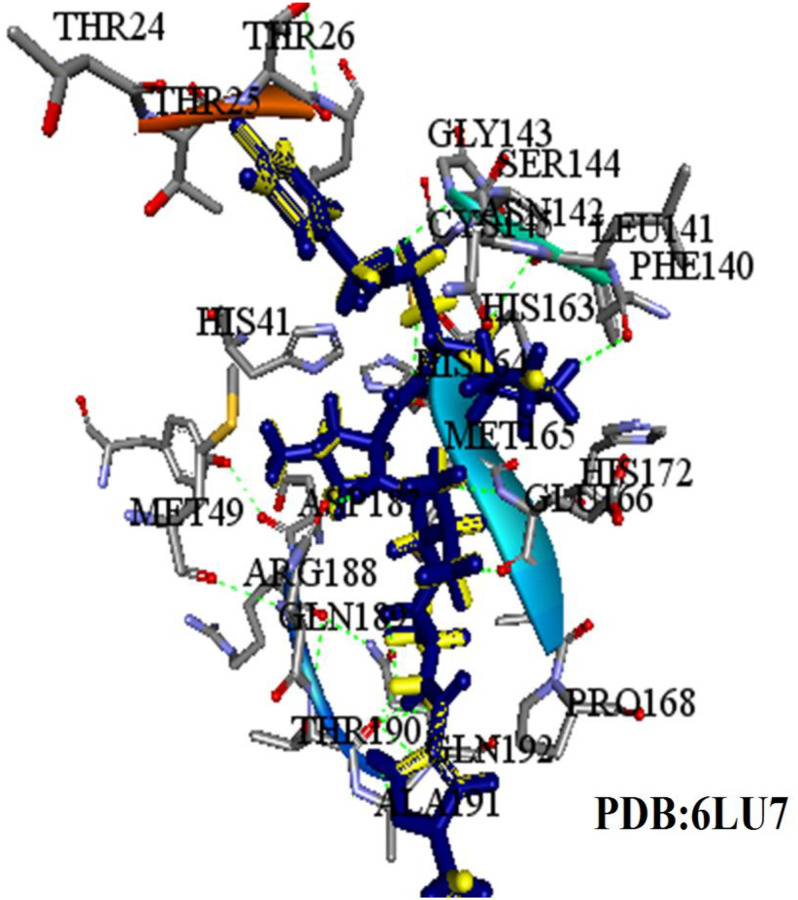
The superimposed image of the N3 inhibitor with 6LU7 protein. Blue represents docked conformation while yellow represents the crystal structure.

Firstly, the validation of the docking procedure was done before carrying out a VS using AutoDock Vina for the selected compounds. A peptide-like inhibitor N3 extracted from a crystallographic COVID-19 main proteinase structure (PDB ID: 6LU7) was re-docked into the same binding pocket. The results showed a similarity between the ligand pose and crystallographic pose (RMSD = 0.88 Å, Figure 3, binding affinity −7.5 kcal/mol). The result indicates that the VS protocol used is reliable, as the RMSD value was below the 2.0 Å threshold value set to evaluate the reliability (Bourne et al., 2003). In the current study, the conserved residues in the binding pocket of COVID-19 3CLprotease have been targeted to block the virus activity.

**FIGURE 4:**
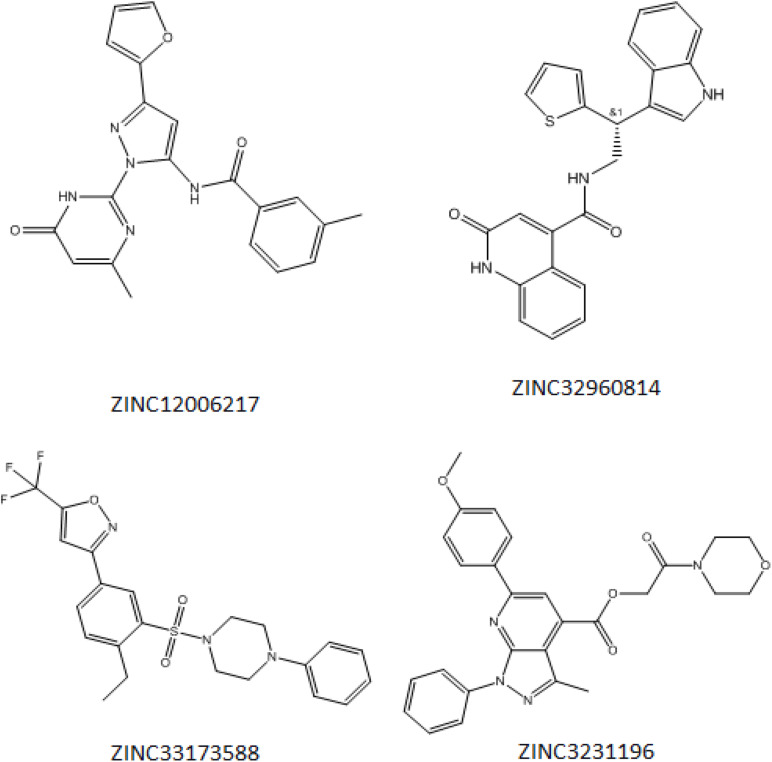
Structure of the four best COVID-19 3CL protease inhibitor candidates with their ZINC Database identification codes (1) ZINC32960814, (2) ZINC12006217, (3) ZINC03231196, and (4) ZINC33173588.

Initially, 2000 ZINC database compounds were virtually screened using AutoDock Vina, further filtered based on Lipinski’s rule of five to evaluate drug likeness of the compounds based on their molecular properties (Lipinski, 2004). Those compounds that violated at least one of rules were removed. The top four ranking ZINC compounds based on AutoDock Vina scores are shown in Figure 4. These compounds had the lowest FEB of the protein-ligand complex amongst all ligands; therefore, these compounds were used for the docking calculation. Their molecular properties are given in Table 1.

**TABLE 1:** Molecular properties of best COVID-19 3CL protease inhibitor candidates from Zinc website.

No	Compounds	xlogP	H-bond donors	H-bond acceptors	Molecular weight(g/mol)	Rotatable bonds
(1)	ZINC32960814	4.633	3	3	413.502	5
(2)	ZINC12006217	3.0854	2	6	375.388	4
(3)	ZINC03231196	3.420	0	8	486.528	6
(4)	ZINC33173588	4.434	0	5	465.497	5
(11)	N3					

The results of VS showed a minimum FEB ranging from −4.3 to −9.5 kcal/mol. A protein-ligand complex with lowest FEB is considered as potential inhibitor (Fornabaio et al., 2004). Consequently, the four compounds that exhibited the lowest FEB were selected as the potential candidates. These compounds are ZINC32960814, ZINC12006217, ZINC03231196, and ZINC33173588 which displayed a minimum FEB of −12.61, −12.32, −12.01, and −11.92 kcal/mol using AutoDock 4.2 and −12.3, −11.9, −11.7, and −11.2 kcal/mol using AutoDock Vina, respectively with the coordinate ligand N3 (shown in Table 2).

**TABLE 2:** FEB values of the best COVID-19 3CL protease inhibitor candidates from Zinc website.

No	Compounds	AutoDock 4.2 FEB (kcal/mol)	AutoDock Vina FEB (kcal/mol)
(1)	ZINC32960814	–12.61	–12.3
(2)	ZINC12006217	–12.32	–11.9
(3)	ZINC03231196	–12.01	–11.7
(4)	ZINC33173588	–11.92	–11.2
(5)	N3	–7.57	–7.5

The results achieved by molecular docking using AutoDock were grouped into clusters of solutions based on the similarity in pose and the free energy of binding, as shown in Table 3 (Smith et al., 2004). The results for compound ZINC32960814 showed that 38 poses adopted a favorable conformation. ZINC12006217 had the largest poses cluster out of 100, whereby it took this pose 44 times, while for the ZINC03231196 adopted 20 times out of 100, likewise, ZINC33173588 is taken this pose 38 times. Table 3 summarizes the cluster analysis showing the total number of clusters and cluster rank, along with the lowest docked energy and range of docking energies. Only the docking mode with the lowest docked energy from this cluster was selected.

**TABLE 3:** Relative cluster ranks and free energies of binding of selected docking modes.

No	Compounds	No of AutoDock cluster	Cluster rank of selected docked structure	Docked free energy range of docked structures	Docked free energy of selected docked structure
(1)	ZINC32960814	38 (100)	5	−12.61 to −11.96	−12.61
(2)	ZINC12006217	44 (100)	3	−12.32 to −9.94	−12.32
(3)	ZINC03231196	20 (100)	2	−12.01 to −10.31	−12.01
(4)	ZINC33173588	25 (100)	5	−11.92 to −9.04	−11.92

**FIGURE 5:**
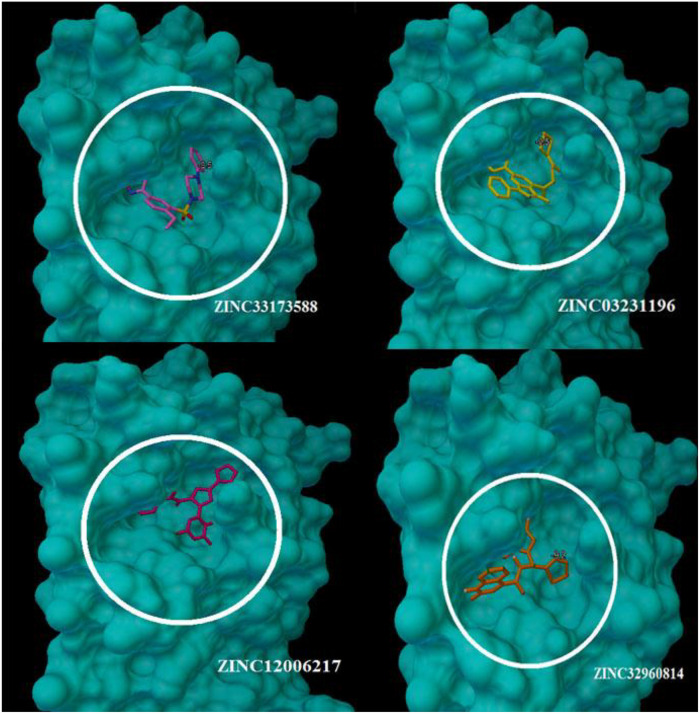
The compounds enfolding at the COVID-19 3CLprotease active site pocket (1) ZINC32960814, (2) ZINC03231196, (3) ZINC12006217, and (4) ZINC33173588.

At the binding pocket, the four compounds were fully wrapped by the amino acids (Figure 5). The interactions analysis between the compounds and amino acids showed that these compounds are located deeply inside the binding pocket of the enzyme in similar shape, indicating that they could be binding covalently with the amino acid residues at this region in 6LU7.

**FIGURE 6:**
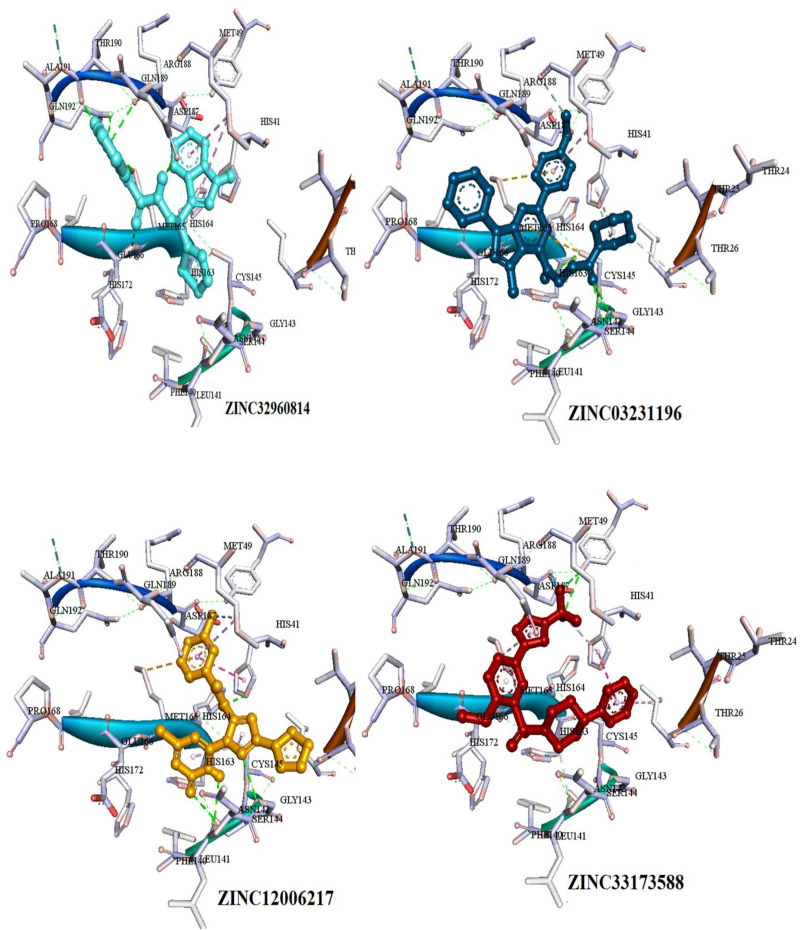
Three dimensional (3D) binding modes of the four compounds present at the COVID-19 3CLprotease binding site represented by stick structure (1) ZINC32960814, (2) ZINC12006217, (3) ZINC03231196, and (4) ZINC33173588A hydrogen bond is indicated by green dotted lines, Pi-sulfur bond is indicated by yellow dotted lines, Pi-Pi T-shipped is indicated by magenta dotted lines, Pi-Alkyl is indicated by purple dotted lines.

The interactions between the docked compounds and the COVID-19 3CLprotease were examined manually using discovery studio visualizer, LigPlot (Laskowski and Swindells, 2011) and AutoDockTool. The extensive interactions between the identified compounds and the amino acids residues that form the binding cavity are shown in Figure 6. These interactions include H-bonding, van der Waals, Pi-alkyl, Pi-Pi T-shaped, and Pi-sulfur interactions.

**FIGURE 7:**
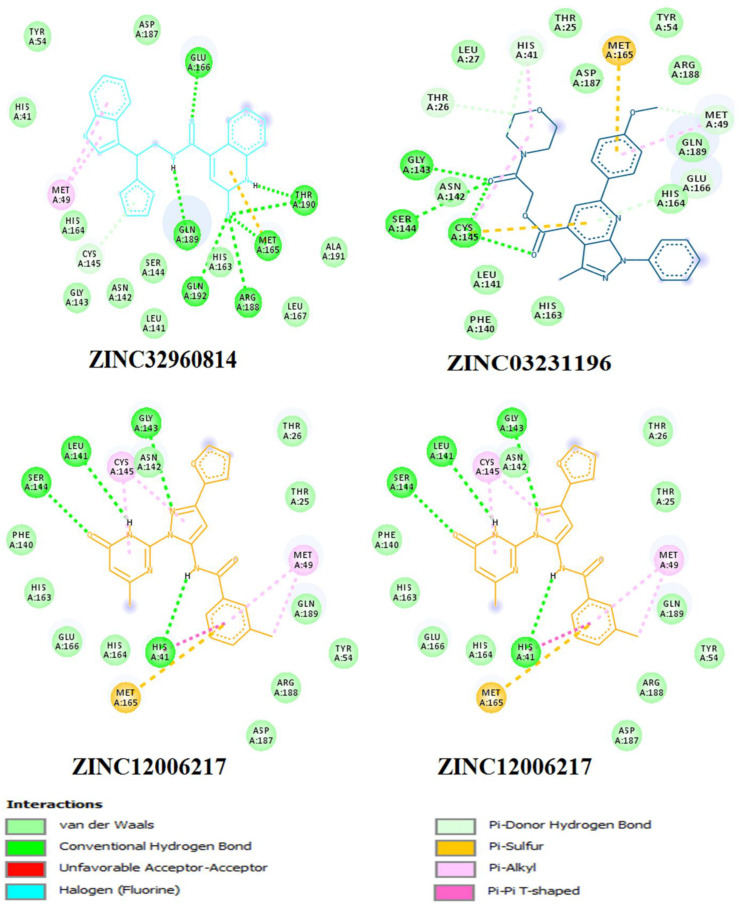
Two dimensional (2D) binding modes of the four compounds present at the COVID-19 3CLprotease binding site represented by stick structure (1) ZINC32960814, (2) ZINC12006217, (3) ZINC03231196, and (4) ZINC33173588.

Among the four selected compounds, ZINC32960814 exhibited the best interactions toward COVID-19 3CLprotease with the lowest FEB of −12.61 kcal/mol, followed by compounds ZINC12006217, ZINC03231196 and ZINC33173588 with FEB of −12.32, −12.01, and −11.92 kcal/mol respectively. The compound, ZINC32960814, displayed seven hydrogen bonds, four between amino acids Gln192, Arg188, Met165, and Thr190 and oxygen atom O2, the other three between Glu166, Gln189, and Thr190 and O1, H10 and N2, respectively. In addition, the compound showed one Pi-sulfur bond between Met165 and benzene ring. Likewise, the amino acid Met49 exhibited two Pi-alkyl bonds with benzene and furan rings on the compounds. The other interactions with the binding pocket were van der Waals between Asp187, Tyr54, His41, His164, Gly143, Ser144, Leu141, Leu167, and Ala191 and the carbon atoms C-1, C-2, C-4, C-22, C-23, C-24, C-13, C-14, and C-15 of the compound. Hydrophobic interactions were displayed with amino acids His163, Phe140, Asn142 at the binding pocket (Table 4 and Figure 7).

**TABLE 4:** Details of binding interactions of the potential four compounds docked into active site of the COVID-19 3CLprotease.

No	Ligands	AutoDock Vina (kcal/mol)	Residue	Type of interactions
(1)	ZINC32960814	−12.61	Glu166, Gln189, Gln192, Arg188, Met165, Thr190, Thr190	H-Bond
			His164, Gly143, Ser144, Leu167, Leu141, Ala191, Tyr54, His41, Asp187	van der Waals
			Met165	Pi-Sulfur
			Met49, Met49	Pi-Alkyl
			His163, Phe140, Asn142	Hydrophobic
(2)	ZINC12006217	−12.32,	His41, Ser144, Leu141, Gly143	H-Bond
			Arg188, Tyr54, Gln189, Thr25, Thr26, Phe140, His163, Glu166, His164	van der Waals
			Met165	Pi-Sulfur
			Met49, Met49	Pi-Alkyl
			His41	Pi-Pi T-shaped
			Cys145, Asn142, Asp187,	Hydrophobic
(3)	ZINC03231196	−12.01	Cys145, Cys145, Ser144, Gly143	H-Bond
			Leu141, Phe140, His163, Lue27, Thr25, Asp187, Tyr54	van der Waals
			His41	Pi-pi T-shaped
			His41, Cys145	Pi-Alkyl
			Met165	Pi-Sulfur
			Gln189, Asn142, Glu166	Hydrophobic
(4)	ZINC33173588	−11.92	Tyr54. Tyr 54	H-Bond
			Arg188, Gln189, His163, Gly143 Ser144 Thr25	Van der Waals
			His41	Pi-pi T-shaped
			Cys145 Lue127	Pi-Alkyl
			Thr26, Asn142, Glu166, Met165	Hydrophobic

Compound ZINC12006217 displayed four hydrogen bonds between amino acids Ser144, Leu141, Gly143, and His41 and atom O2, N4, N2, and N1, respectively. Amino acid Met165 formed Pi-sulfur bond with the benzene ring. Four Pi-alkyl interactions were formed, two between Cys145 and N27 atom on the furan ring while another two were formed between Met49 with C-1 and benzene ring. Likewise, van der Waals interactions were noticed between amino acids namely Arg188, Tyr54, Gln189, Thr25, Thr26, Phe140, His163, Glu166, His164, and carbon atoms C-1, C-2, C-3, C-23, C-15, C-16, C-22, C-23, C-23, and C-25 of the compound, while hydrophobic interactions showed between amino acids and atoms Cys145 with atoms C-12, N3, Asn142 with C-20, Asp187 with C-1, C-3 and Thr26 with C-19(Table 4 and Figure 7). The third compound ZINC03231196 exhibited four hydrogen bonds, two of them formed between the two oxygen atoms O11 and O15 and Cys145, while the other two between O11 and two amino acids namely Gly143 and Ser144. In addition, Pi-alkyl interactions were formed between His41 and Cys145 and morpholine ring, while Met49 formed another Pi-alkyl with the second benzene ring on the compound. Likewise, a Pi-sulfur bond was formed between benzene rings and Cys145 and Met165. Van der Waals interactions were also noticed between amino acids Leu141, Phe140, His163, Lue27, Thr25, Asp187, Tyr54 and C-30, C-31,C-32, C-3, C-4, C-5, C-8, C-9, and C-23. Hydrophobic interactions were formed between the compound and amino acids Gln189, Asn142, Glu166 (Table 4 and Figure 7). Compound ZINC33173588 exhibited two hydrogen bonds between the two fluorine atoms F13 and Tyr 54, as well as two halogen bonds (fluorine bonds) with Asp187. Likewise, His41 formed Pi-Pi T-shaped bond with first benzene ring, two Pi-alkyl bonds were formed between amino acids Cys145, His41 and morpholine ring, another Pi-alkyl interaction was formed between Met165 and furan ring, Pi-donor hydrogen bond was also noticed with C-23. Van der Waals interactions were formed between the amino acids Arg188, Gln189, His163, Gly143, Ser144, Thr25 and carbon atoms C- 23, C-25, C-26, C-6, and C- 5, while hydrophobic interaction was showed with the amino acids Thr26, Asn142, Glu166, Met165 (Table 4 and Figure 7).

Many interactions such as hydrogen bonding, van der Waals, hydrophobic and Pi-Pi interactions occurred between the identified compounds and the essential amino acids at the binding pocket, especially His41 and Cys145, where the amount and type of bonding formed revealed high affinity with the COVID-19 3CLprotease.

For the comparison purpose, ten clinically used drugs obtained from DrugBank (Wishart et al., 2006), five known as antivirus and others as anti-malaria were introduced to the VS, their results of FEB ranged from −8.7 to −6.1 Kcal/mol (Table 5).

**TABLE 5:** FEB values of 10 FDA approved Antivirus and Anti-malaria drugs.

No	Code	Name	FEB kcal/mol
(1)	DB00220	Nelfinavir	−8.7
(2)	DB00224	Indinavir	−7.8
(3)	DB00194	Vidarabine	−6.6
(4)	DB00238	Nevirapine	−6.4
(5)	DB00198	Oseltamivir	−6.4
(6)	DB13132	Artemisinin	−7.5
(7)	DB01190	Clindamycin	−7.5
(8)	DB00908	Quinidine	−7.0
(9)	DB00207	Azithromycin	−7.0
(10)	DB00608	Chloroquine	−6.1

**FIGURE 8:**
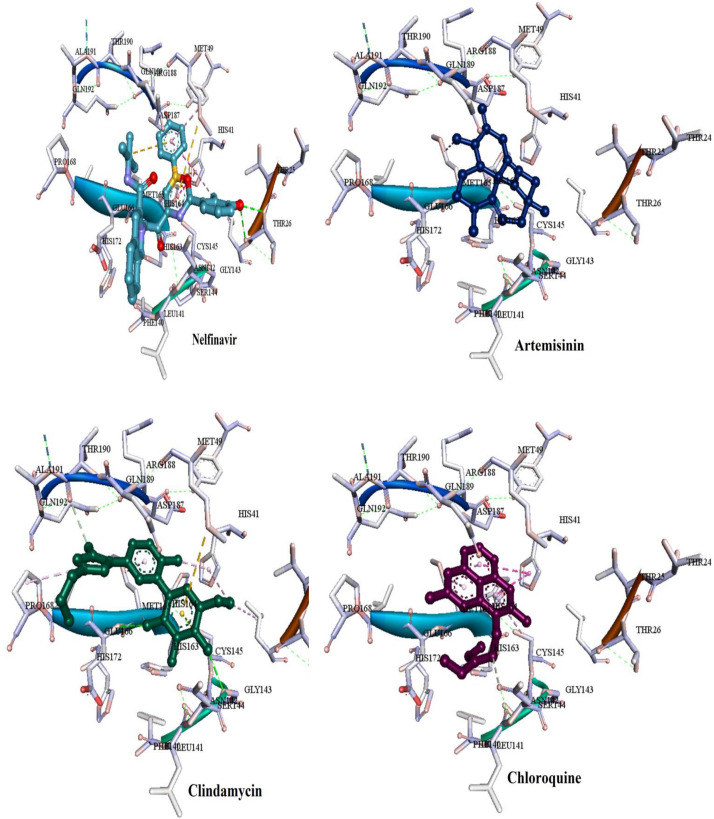
Binding modes of the FDA approved drugs present at the COVID-19 3CLprotease binding site represented by ball and stick (one antiviral drug, nelfinavir and three antimalarial drugs, artemisinin, clindamycin, and chloroquine).

Most of the used FDA drugs showed different interactions with the target protein of the binding pocket. Among antiviral drug, nelfinavir, which exhibited lowest FEB of −8.7 kcal/mol, formed few molecular interactions, one hydrogen bond with Thr26, two sulfur bonds with Met165 and Met49 and Pi-alkyl formed with Leu27, His41 and Met49. Besides, van der Waals interactions were formed with residues Glu166, Leu141, His163, Asp1187, Arg188, Ser144, and Gln189 (Figure 8). On the other hand, for the anti-malaria artemisinin and clindamycin exhibited similar FEB of −7.5 kcal/mol. Artemisinin exhibited only two types of interactions, Pi-alkyl with Met165 and van der Waals with Gln189, Asp187, Arg188, His164, His41, Met49, Leu27, and Cys145, while clindamycin showed two H-bonds with Gly143 and Glu166, and Pi-sulfur bonds with residues Met49 and Cys145. In addition, clindamycin had three pi-alkyl interaction formed with Pro168, His41, and Leu27, and van der Waals interactions formed with the amino acids Thr25, Asp187, Arg188, Glu189, Gln192, Leu141, Ser144, and Asn142 (Figure 8). For the well-known anti-malaria drug, chloroquine, recently different studies have brought attention to possibilities of using this drug in the treatment of coronavirus SARS-CoV-2 infection (Gao et al., 2020; Li and De Clercq, 2020; Touret and de Lamballerie, 2020; Lee et al., 2020; Wang et al., 2020). The docking result of chloroquine showed FEB of −6.1 kcal/mol, forming only three types of interactions at the binding pocket, Pi-alkyl interaction with Cys145 and Met165, Pi-Pi T-shaped interaction with His41 As well as van der Waals interaction with amino acids Asp187, Arg188, Gln189, Glu166, His164, and Gly143 (Figure 8).

From the above findings, it was found that the four identified compounds from the ZINC database showed high affinity and good binding interactions. These compounds were inhibitor targets for the catalytic dyad Cys145 and His41 along with the other amino acids residues at the binding pocket, this ability to interact with COVID-19 3CLprotease offers additional benefits of inhibiting the virus activity. Moreover, these compounds show an advantage over the known FDA drugs in terms of types and amount of interactions and FEB that make them potential for COVID-193 CLprotease inhibition.

## Conclusion

In the present study, VS and molecular docking molecular interaction analysis were successfully applied in identification of inhibitors for COVID-19 3CLprotease. The four compounds namely ZINC33173588, ZINC03231196, ZINC12006217, and ZINC32960814 exhibited high affinity with the 3CLpro binding pocket of COVID-19. The free energy of binding (FEB) were −12.3, −11.9, −11.7, and −11.2 kcal/mol while AutoDock Vina scores were −12.61, −12.32, −12.01, and −11.92 kcal/mol, respectively. The finding suggests that the four compounds were strongly bound to the 3CL-protease of COVID–19 in comparison with the FDA approved clinically used drugs. The top docking hits passed the Lipinski rule of five and likely to be orally active drug. Sequence alignment showed the similarity between SARS-CoV and COVID-19 catalytic dyad residues Cys145 and His41. The obtained results revealed that the interactions of the compounds with the conserved catalytic dyad amino acids Cys145 and His41 was closer in comparison to that of ligand N3 and FDA drugs. Application of VS and molecular docking could significantly decrease the cost of the drug synthesis and production, subsequently, and provided evidence for interactions of the identified compound with the target COVID-19 3CLprotease. Experimental studies (*in vivo*) are needed to confirm the findings and to investigate their effects in COVID-19 using an appropriate animal model.

## Data Availability Statement

The datasets presented in this study can be found in online repositories. The names of the repository/repositories and accession number(s) can be found below: http://www.wwpdb.org/.

## Author Contributions

AA involved in the conceptualization, design, analysis of data, performed all computational studies, and wrote the manuscript. VM reviewed, edited, and made corrections of the manuscript.

## Conflict of Interest

The authors declare that the research was conducted in the absence of any commercial or financial relationships that could be construed as a potential conflict of interest.
